# The Endothelium, A Protagonist in the Pathophysiology of Critical Illness: Focus on Cellular Markers

**DOI:** 10.1155/2014/985813

**Published:** 2014-04-01

**Authors:** Sabrina H. van Ierssel, Philippe G. Jorens, Emeline M. Van Craenenbroeck, Viviane M. Conraads

**Affiliations:** ^1^Department of Critical Care Medicine, Antwerp University Hospital (UZA), University of Antwerp (UA), Wilrijkstraat 10, 2650 Edegem, Belgium; ^2^Department of Internal Medicine, Antwerp University Hospital (UZA) and University of Antwerp (UA), Wilrijkstraat 10, 2650 Edegem, Belgium; ^3^Laboratory of Cellular and Molecular Cardiology, Antwerp University Hospital (UZA), University of Antwerp (UA), Wilrijkstraat 10, 2650 Edegem, Belgium; ^4^Department of Cardiology, Antwerp University Hospital (UZA), University of Antwerp (UA), Wilrijkstraat 10, 2650 Edegem, Belgium

## Abstract

The endotheliumis key in the pathophysiology of numerous diseases as a result of its precarious function in the regulation of tissue homeostasis. Therefore, its clinical evaluation providing diagnostic and prognostic markers, as well as its role as a therapeutic target, is the focus of intense research in patientswith severe illnesses. In the critically ill with sepsis and acute brain injury, the endothelium has a cardinal function in the development of organ failure and secondary ischemia, respectively. Cellular markers of endothelial function such as endothelial progenitor cells (EPC) and endothelialmicroparticles (EMP) are gaining interest as biomarkers due to their accessibility, although the lack of standardization of EPC and EMP detection remains a drawback for their routine clinical use. In this paper we will review data available on EPC, as a general marker of endothelial repair, and EMP as an equivalent of damage in critical illnesses, in particular sepsis and acute brain injury. Their determination has resulted in new insights into endothelial dysfunction in the critically ill. It remains speculative whether their determination might guide therapy in these devastating acute disorders in the near future.

## 1. Introduction

The endothelium forms the inner layer of blood and lymphatic vessels [1, 2]. Besides its mere role as a barrier between blood and tissue, the endothelial cell layer displays a myriad of physiological functions. Integrity of the endothelium is required for adequate deliverance of oxygen and nutrition to tissue and the migration of blood cells. Furthermore it plays a central role in coagulation and fibrinolysis, it regulates vascular tone and the formation of new blood vessels. As such the endothelium is a key regulator of homeostasis, for which continuous interaction with its environment is crucial. Its importance in the pathophysiology of not only cardiovascular, but also inflammatory and malignant diseases, is increasingly recognized.

The clinical evaluation of the endothelium has been thwarted by its location at the inner side of the vessels. The growing interest in the endothelium as a central player in numerous diseases has stimulated the development of a multitude of new circulating markers and in vivo evaluation techniques [1].

In this review we will focus on critically ill patients with sepsis and acute brain injury, both devastating conditions seen frequently in the intensive care unit. Sepsis and acute brain injury are characterized by secondary complications, that is, multiorgan failure and cerebral ischemia, respectively, which have enormous impact on outcome. Vascular dysregulation and endothelial dysfunction play a central role herein. As such, markers of endothelial dysfunction are of potential interest in determining prognosis.

## 2. Enumeration of Circulating Cellular Markers of Endothelial Dysfunction

During the last two decades cellular markers of endothelial repair and damage have emerged as potential noninvasive candidates for functional evaluation of the endothelium. In this overview, we highlight the role of endothelial progenitor cells (EPC) as a marker of endothelial repair and endothelial microparticles (EMP) as a measure for endothelial damage. We will briefly discuss their methods of detection. For thorough discussion on these matters we refer to recently published reviews [3–7].

Endothelial progenitor cells originate from the bone marrow and can differentiate into mature endothelial cells [3, 8]. In situations of ischemia and in case of inflammation, EPC repair damaged endothelium and help in creating capillary networks, in a direct and paracrine fashion [9]. Several humoral factors are implicated in their mobilization, differentiation, and homing such as vascular endothelial growth factor (VEGF), granulocyte macrophage colony forming factor, stromal derived factor 1*α* (SDF 1*α*), erythropoietin (EPO), amongst others [10]. Despite a multitude of papers published on EPC in various diseases, their definition remains a point of debate. The confusion on EPC definitions originates from the various techniques used for their detection having poor intermethod agreement (different types of cell culture techniques and flow cytometry) [3, 4, 11]. In cell culture techniques we discriminate two types, that is, short-term culture identifying early outgrowth EPC and long-term cultures isolating truly proliferating cells with endothelial fate [3, 4]. Since the first rather identifies hematopoietic cells involved in angiogenesis and the last are very elaborative, long-term cultures up to 30 days necessitating large amounts of blood and resulting in low colony counts, flow cytometry is at this moment the preferred technique for their detection in clinical studies. However there is a lack of any specific phenotypic marker for EPC to use in flow cytometry. Asahara et al. were the first to describe putative EPC, and they used a combination of CD34, a hematopoietic and progenitor cell marker, and flk-1/KDR, a receptor for VEGF important for homing of EPC and expressed on endothelial cells [8]. Both markers, however, are rather aspecific and as such are also expressed on mature circulating endothelial cells. For this reason Peichev et al. added CD133, a stem cell marker to better differentiate true EPC [12]. A drawback of using these triple positive cells, as circulating marker of endothelial function, is that their number is so low that enumeration becomes less reliable [3]. Furthermore it has been shown that these cells do develop into hematopoietic and not endothelial colonies [13]. EPC defined as CD34 and KDR positive cells have been most widely evaluated in clinical studies and have proven to be implicated in angiogenesis and endothelial repair in vivo [4, 9, 14]. Hence our research group prefers to use these cells as markers of endothelial repair, keeping in mind that this is a heterogeneous group of cells possessing an overlapping phenotype with endothelial cells and hematopoietic progenitors [6].

Endothelial microparticles (EMP) originate through vesiculation of the endothelial cell membrane upon cell activation, damage, or apoptosis [38]. EMP are membrane particles smaller than 1 µm, which contain oxidized phospholipids and proteins characteristic of endothelial cells. Surface antigens vary with the microparticle generating process; CD31+, CD105+, and Annexin V+ EMP are generated mainly during apoptosis, while CD62E, CD54, and CD106 expression are mostly seen when E are released upon activation [39, 40]. For their detection flow cytometry is the mainly used and mostly studied technique [41]. It has been shown that preanalytical and analytical heterogeneity amongst various research groups has led to differing results [38, 41, 42]. At this moment efforts are made for analytical and preanalytical standardization for flow cytometric detection of microparticles [43, 44]. Another difficulty for the use of EMP as biomarker in the critically ill is the possible interference with lipid-rich solutions [45]. The use of propofol and total parenteral nutrition in these patients could lead to secondary lipid accumulation negatively influencing the number of microparticles detected by flow cytometry. EMP are increasingly used as a marker of endothelial damage, especially in cardiovascular disorders, but growing evidence also indicates that EMP have an important modulating role in inflammation, coagulation, and vascular function [5, 38].

Multiparameter analysis for the evaluation of endothelial function is emerging as a valuable ex vivo tool for assessment of endothelial function [46], in addition to its potential to further unravel the pathophysiology of endothelial disruption in several disease conditions [7].

## 3. The Endothelium in Sepsis: The Orchestrator of Organ Failure

In one of four patients hospitalized at the intensive care unit severe sepsis is the reason for admission [47, 48]. Sepsis is defined as the systemic inflammatory response syndrome to an infection [49]. It is a devastating disorder that can progress to severe sepsis with the development of organ dysfunction, septic shock when hypotension is unresponsive to fluid resuscitation, and eventually multiorgan failure and death [49]. These stages of severity form a continuum in which patients evolve during their disease and treatment. The chance of survival decreases with the progression of the sepsis syndrome over this continuum. Hospital mortality in sepsis varies between 14 and 45% in Europe [47, 48]. Despite important advances in microbiological and supportive therapy, mortality has only slightly improved during the last decades [47]. Organ failure is the major cause of death in sepsis patients [50]. This is further supported by the finding that the number of organs that failed correlates with short-term mortality [51] and that the therapeutic improvement of organ failure early in sepsis improves survival [52].

### 3.1. Endothelial Function in Sepsis

The pathophysiology of sepsis is complex. Being multifactorial and heterogeneous among patients is two of the main characteristics of sepsis [53]. Sepsis is caused by a systemic maladaptive response of the host to an invading microorganism. Under normal conditions, infection triggers a local inflammatory reaction associated with an antiinflammatory response, local activation of the coagulation process together with a systemic acute phase and neurohumoral response. All these reactions are finely tuned with the purpose of containing the infection with minimal damage. The complex interaction of these responses leading to the conquest of the infection in one case could derail and lead to sepsis in others. The exact factors leading to sepsis are not completely understood but are host and microorganism dependent. The endothelium has a central position in orchestrating both the physiologic and pathological host response to infection due to its regulation of cellular permeability, coagulation, and vascular blood flow [54].

In the development of distant organ dysfunction, macro- and microvascular dysfunction play an important role. Macrovascular dysfunction during sepsis constitutes of 2 major effects: hyperdynamic shock due to hypovolemia caused by venous and arterial vasodilation at the macrovascular level and capillary leak and cardiac depression [55]. On the other hand it has been shown that tissue hypoperfusion remains despite normalization of macrocirculatory derangements, underlining the importance of additional microvascular derangements and mitochondrial dysfunction in sepsis [56, 57]. At microvascular level there is heterogeneity in flow, stopped flow, and decreased density of perfused capillaries [56]. As such, in sepsis the microcirculation is unable to adequately regulate microvascular perfusion to local oxygen demand.

**Figure 1:**
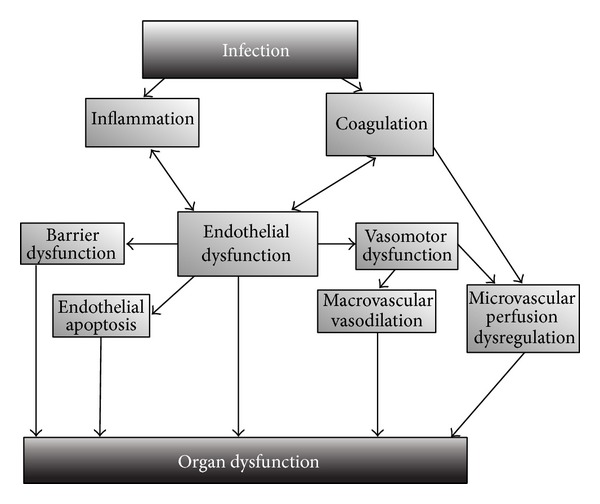
Endothelial dysfunction in sepsis. In sepsis the reaction that has the aim of containing the infection derails and leads to a proinflammatory, procoagulant situation and endothelial dysfunction, finally resulting in the development of organ failure.

The endothelium is a key component in the development of these macro- and microcirculatory disturbances in sepsis (see Figure 1). Activation of the endothelium leads to a procoagulant and proinflammatory condition, a disrupted barrier and an abnormal vascular tone [2]. In sepsis there is a direct destruction of the endothelial barrier [2, 58], and an increased amount of circulating endothelial cells has been shown in patients with septic shock [59]. The vasomotor regulation is also hampered in sepsis. More in particular there is an imbalance between vasodilator and vasoconstrictor signaling molecules leading to an impaired vasomotor tone. Despite increased concentration of circulating catecholamines in sepsis there is a decreased vascular response to these factors [60]. On the other hand a disturbed endothelial mediated vasodilation has also been shown at macrovascular and microvascular level [61–64].

Evaluating circulating endothelial markers in patients with sepsis has evolved from circulating endothelial adhesion-molecules to cellular markers of endothelial repair and damage.

### 3.2. Endothelial Progenitor Cells in Sepsis

Several groups have investigated EPC in sepsis but their role has not yet been unequivocally defined [15–22] (see Table 1). Observational studies found an increased percentage of circulating EPC enumerated by flow cytometry in highly selected patients with sepsis [15, 19, 20], while experimental studies, that is, the administration of LPS in healthy volunteers and a MODS model in pigs showed a decreased number [17, 18]. At our own center, we found a decreased absolute number of EPC in a heterogeneous group of severe sepsis patients compared to healthy volunteers [22]. Furthermore, while Becchi et al. found an increased number of EPC in severe sepsis versus sepsis patients, Rafat et al. found a positive correlation between EPC number and survival [15, 20]. Our data are in line with the last findings, with lower numbers of absolute EPC in patients with increasing sequential organ failure assessment (SOFA) score, a measure of severity of organ failure, during the first week after sepsis. Differences in study population, expressing results as percentage of peripheral blood mononuclear cells (PBMC) (which are decreased in sepsis) versus absolute numbers; methodology (isolated PBMC versus whole blood) and the different phenotypes studied can explain these opposing findings. Despite these contradictory findings, the functional impairment of EPC seems indisputable in sepsis [16–19, 22]. All studies, case-control and experimental, describe decreased proliferative or migratory capacities of EPC [16, 17, 19, 21, 22]. Data on the exact role of EPC in vascular repair during sepsis are scarce. Lam et al., for example, showed that EPC transplantation in a rabbit ARDS model decreased endothelial dysfunction, maintained the alveolocapillary membrane, and reduced inflammation [65]. As mentioned before, numerous humoral factors influence EPC mobilization, differentiation, and homing; therefore, EPC are an important therapeutic target to stimulate endothelial repair in sepsis. Several therapeutic strategies that focus on sepsis-related endothelial dysfunction have been shown to influence EPC [66], for example, statins, shown to increase EPC number and ameliorate their functional capacity, that is, decreasing senescence and improving proliferation [67, 68].

### 3.3. Endothelial Microparticles in Sepsis

Several studies, case-control human studies as well as animal models, have explored EMP in sepsis, with differing results (see Table 2) [22, 23, 25–27, 29]. EMP were found to be increased in patients with sepsis by some research groups [25, 26, 29], while others found a decreased or equal number [22, 23]. These inconsistent results may result from a lack of pre- and analytical standardization of microparticle (MP) detection, the different phenotypes studied, and differences in study population. In contrast to the interpretation in cardiovascular diseases, where an elevation of EMP is considered a marker of endothelial dysfunction, the number of EMP is positively related to survival and inversely correlated with the SOFA-score in patients with sepsis [29]. Since it is becoming more and more clear that microparticles are more than simple markers of endothelial damage or activation, their interpretation as marker of endothelial dysfunction is less unambiguous. As such it has been shown that the general pool of MP in septic patients is protective against vascular hyporeactivity in vitro, increasing the response to 5-HT in vitro while not affecting endothelium-dependent vasodilation [25]. Mortaza et al., on the other hand, found that injection of MP from septic rats induced vasodilatory shock in healthy animals [24]. MP also have been implicated in hypercoagulability and inflammation [23, 26, 29]. Finally Pérez-Casal et al. found increased numbers of MP bearing endothelial protein C receptor (EPCR) of endothelial and monocytic origin in patients treated with recombinant protein C [28]. These MP decreased apoptosis and reduced permeability in endothelial cells in an APC dependent way, a confirmation of earlier in vitro findings [69]. At this moment the knowledge on EMP functions in sepsis is too scarce to clarify their role in the development of organ failure.

## 4. The Endothelium as Key Player in Secondary Cerebral Ischemia after Acute Brain Injury

Acute brain injury, more in particular subarachnoid hemorrhage (SAH) and traumatic brain injury (TBI), is devastating neurological events which have an important socioeconomical impact. The development of secondary cerebral ischemia is an important prognostic factor in both SAH and TBI [70–72]. It develops in 8–12% and 20–30% of patients after TBI and SAH, respectively, mostly within the first 2 weeks after the insult [70, 71, 73, 74].

### 4.1. Endothelial Function and Secondary Cerebral Ischemia after Subarachnoid Bleeding

**Figure 2:**
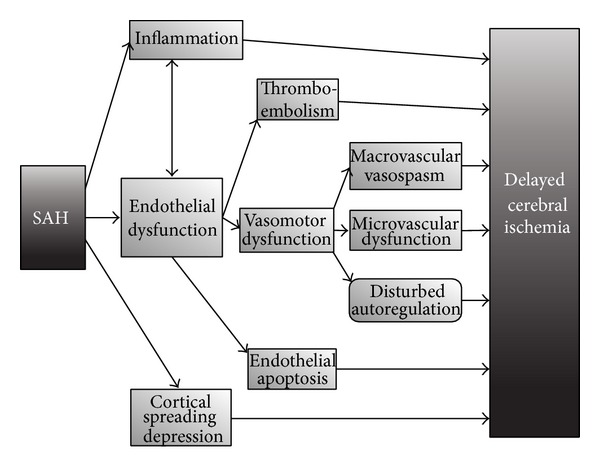
Endothelial dysfunction in SAH. SAH: subarachnoid hemorrhage. In subarachnoid hemorrhage, the development of delayed cerebral ischemia is a multifactorial process in which besides macrovascular vasospasm; thromboembolism, disturbed autoregulation, microvascular dysfunction, and cortical spreading depression are involved. Endothelial dysfunction is a key factor in the development of these processes. It is not clarified yet if local and general inflammation are causal factors or bystanders in the development of secondary ischemia.

In SAH the concepts of delayed cerebral ischemia (DCI) and cerebral vasospasm have been well studied and clearly defined (see Figure 2) [75]. While previously macrovascular cerebral vasospasm was thought key for the development of DCI, it is now accepted to be a multifactorial process of which the exact underlying mechanisms are not yet completely unraveled [75]. As such it has been repetitively shown that macrovascular vasospasms are not a condition sine quo non to develop DCI, and on the other hand not all vasospasms will lead to the development of DCI [74]. Other mechanisms such as microvascular dysfunction, disturbed autoregulation, thromboembolism, and cortical spreading depression have been implicated in its development [76–78]. The endothelial function, in all its aspects, is a crucial factor in these proposed mechanisms. It plays a central role in the formation of microthrombi by regulating vasoconstriction and expressing of P-selectin [79]. Furthermore it has been shown that cerebral vascular reactivity and cerebral autoregulation are disturbed after SAH [77, 80]. The endothelium plays an important role in modulating vascular tone. As such both endothelial derived vasodilators (e.g., NO) and vasoconstrictors (e.g., endothelin) are important in the development of macrovascular cerebral vasospasm and in microvascular dysfunction [81, 82]. Moreover, cerebral endothelial cell apoptosis has been documented after experimental SAH [83]. The role of inflammation in the development of ischemia is not clarified yet, but the endothelium is important in the regulation of diapedesis of leucocytes and local inflammation [81, 84].

### 4.2. Endothelial Function and Secondary Ischemia after Traumatic Brain Injury

**Figure 3:**
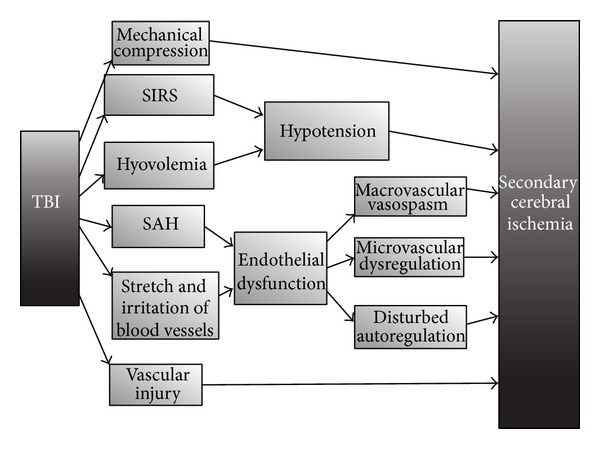
Development of delayed cerebral ischemia after traumatic brain injury. SAH: subarachnoid hemorrhage; SIRS: systemic inflammatory response syndrome; TBI: traumatic brain injury. In traumatic brain injury the exact pathophysiology of secondary ischemia is not completely clarified. Besides cerebral mechanisms, extracerebral processes are also involved such as hypotension. On the other hand the endothelium seems to be a central player in its development.

In traumatic brain injury (TBI), on the other hand, the concept of posttraumatic cerebral ischemia is less well studied and understood. This can be explained by the fact that patients with TBI are a very heterogeneous group and that besides the primary cerebral injury other extra-cerebral processes may cause secondary damage [70, 71, 85]. The mechanisms involved are mechanical compression, hypotension, direct vascular injury, thromboembolism, and posttraumatic cerebral vasospasm; moreover, distinguishing these is difficult (see Figure 3). The appearance of posttraumatic cerebral vasospasms has been related to the presence of traumatic subarachnoid blood but has also been reported in the absence of a traumatic SAH [86]. These findings suggest that besides the mechanisms important in the development of vasospasm after spontaneous SAH, other processes are involved after TBI, such as direct stretching and mechanical irritation [86]. Furthermore the relation between cerebral vasospasm and the development of secondary ischemia is still a point of debate, and there are only few prospective studies on this matter [86, 87]. Besides macrovascular changes, the microvasculature is also involved. As such in animal experiments diffuse loss of microvasculature networks and capillary density after TBI were found [88]. Increased VEGF expression, indicating a possible role for neovascularization [88], and impaired cerebral endothelium-dependent cerebral vascular responses have also been documented [89].

### 4.3. Endothelial Progenitor Cells after Acute Brain Injury

Until now research on markers of endothelial function in SAH and TBI has mostly focused on circulating endothelial adhesion molecules and markers of endothelial activation [90–92], both of which are increased in patients developing secondary cerebral ischemia. Endothelial progenitor cells show a biphasic response after traumatic brain injury; after an initial decrease they peak 7 days after the insult (see Table 3) [31]. Furthermore they have been associated with an improved outcome after TBI [32]. In patients with cerebral aneurysm a decreased number of EPC also has been shown, possibly related to patients' risk factors (e.g., smoking and hypertension) [34]. Our group also enumerated EPC in patients with SAH and TBI and confirmed the finding of a decreased number of EPC initially after the insult. (van Ierssel S.H., unpublished results) publication) Furthermore an impaired functional capacity of EPC was seen [30, 34]. After endovascular coiling of ruptured aneurysm there is a rapid increase of EPC with a peak at 14 days after rupture [33]. At this moment, we are not aware of studies on the relation between EPC and DCI or posttraumatic cerebral ischemia. The exact role of EPC in vascular repair after acute brain injury has not been studied yet. In a rat model of traumatic brain injury Wang et al. looked at the role of atorvastatin [93]. They found an increased number of EPC and enhanced cerebral angiogenesis, together with an improved functional outcome in treated rats. These results again show the importance of EPC as a possible therapeutic target.

### 4.4. Endothelial Microparticles in Acute Brain Injury

Few researchers have looked at the evolution of endothelial microparticles after SAH and TBI (see Table 4) [35–37]. They all found an increased number of EMP after acute brain injury, in line with the common use of EMP as markers of endothelial damage [38]. With regard to the development of secondary ischemia, the relation seems more ambiguous. While Lackner et al. found an increased number of CD105+ Annexin+ EMP early after the insult, Sanborn et al. found a decreased number of CD146+ EMP at Day 1 in patients developing DCI [35, 37]. The different populations that were studied can explain these opposing results, as well as the variable pre- and analytical methods used and the variances in phenotype of EMP studied. At this moment there are no data available on the exact functional role of EMP after acute brain injury.

## 5. Conclusion

The endothelium seems to be a central actor in the development of organ failure in sepsis and secondary ischemia after acute brain injury, as illustrated here for SAH and TBI. The exact role of markers of repair (EPC) and injury (EMP), however, needs further clarification. Nevertheless the importance of both organ failure and secondary ischemia in the prognosis of these devastating disorders explains the craving for adequate prognostic and therapeutic clues and hence the interest in the endothelium makes common sense.

## Figures and Tables

**Table 1:** Overview endothelial progenitor cells in sepsis.

Study group	Study type	Phenotype EPC	Main findings
Becchi et al. [15]	Case-control sepsis (*n* = 24)	CD34+ KDR+ in isolated PBMC CD34+ KDR+ CD133+ in isolated CD34+ cells	(i) Increased % EPC the first 72 h after sepsis (ii) EPC severe sepsis ≫ sepsis

Cribbs et al. [16]	Case-control sepsis (*n* = 86)	CFU-EPC	(i) Decreased CFU-EPC in sepsis (ii) Inversely associated with SOFA

Luo et al. [17]	MODS model in pigs (*n* = 20)	CD133+ CD34+ in WB CFU-EPC Migration to VEGF	Decreased EPC, CFU-EPC and migratory function in MODS

Mayr et al. [18]	LPS in healthy volunteers (*n* = 32)	CD34+ KDR+ CD133+ EPC in WB CFU-EPC	(i) Decrease in EPC number with nadir at 6 h post LPS (ii) Decreased CFU-EPC nadir 4 h after LPS

Patschan et al. [19]	Case-control sepsis (*n* = 40)	KDR+ CD133+ in isolated PBMC CFU-EPC	(i) Increased % EPC in sepsis (ii) Decreased CFU-EPC in sepsis

Rafat et al. [20]	Case-control sepsis (*n* = 32)	CD34+ KDR+ CD133+ in isolated PBMC	(i) Increased % EPC in sepsis (ii) Lower % EPC in nonsurvivors

Schlichting et al. [21]	Case-control severe sepsis (*n* = 18)	CFU-EPC	No difference

van Ierssel et al. [22]	Case-control severe sepsis (*n* = 30)	CD34+ KDR+ in WB Migration to SDF-1*α* and VEGF	(i) Decreased absolute number (ii) Decreased migratory capacity (iii) Impending organ dysfunction the first week was associated with lower EPC and a trend to impaired migration

CFU-EPC: EPC colony forming units; EPC: endothelial progenitor cells, LPS: lipopolysaccharides; MODS: multiorgan dysfunction syndrome; PBMC: peripheral blood mononuclear cells; SDF-1*α*: stromal derived factor 1 *α*; SOFA: sequential organ failure assessment; VEGF: vascular endothelial growth factor; WB: whole blood.

**Table 2:** Overview endothelial microparticles in sepsis.

Study group	Study design	Detection method	Phenotype EMP	Main findings
Joop et al. [23]	Case-control MODS and sepsis (*n* = 9)	Flow cytometry isolated MP frozen samples	CD62E+/ Annexin V+ CD144+/ Annexin V+	(i) Lower number CD62E+ EMP (ii) Unchanged number CD144+ EMP

Mortaza et al. [24]	Rat cecal ligation and puncture model	Flow cytometry PFP	CD54+/ Annexin V+	(i) Unchanged EMP in sepsis (ii) Septic MP caused vasoplegic shock in healthy rats

Mostefai et al. [25]	Case-control sepsis (*n* = 36) mouse model: injection of septic MP	Flow cytometry PFP frozen samples	CD146+	(i) Increased EMP in sepsis (ii) Septic MP induced increased responsiveness to vasoconstrictors in aortic rings

Nieuwland et al. [26]	Case-control meningococcal sepsis (*n* = 7)	Flow cytometry isolated MP	CD62E+/ Annexin V+	Nonsignificant increase in sepsis

Ogura et al. [27]	Case-control severe SIRS (*n* = 28, sepsis = 12)	Flow cytometry PRP	CD54+ CD31+	EMP increased in sepsis

Pérez-Casal et al. [28]	Case control study of APC treated sepsis patients	Flow cytometry isolated MP	CD13+ EPCR+	Increased CD13+ EPCR+ MP

Soriano et al. [29]	Case control severe sepsis (*n* = 35)	Flow cytometry PPP	CD31+ CD42b−	(i) EMP higher in severe sepsis (ii) EMP higher in survivors (iii) Negative correlation with SOFA on D2 and D3

van Ierssel et al. [22]	Case-control severe sepsis (*n* = 26)	Flow cytometry PPP	CD31+ CD42b−	Unchanged number of EMP versus healthy controls

EMP: endothelial microparticles; MODS: multiorgan dysfunction syndrome; MP: microparticle; PFP: platelet free plasma; PPP: platelet poor plasma; PRP: platelet rich plasma; SOFA: sequential organ dysfunction assessment.

**Table 3:** Overview endothelial progenitor cells in acute brain injury, SAH, and TBI.

Study group	Study type	Phenotype EPC	Main findings
Liang et al. [30]	Case-control unruptured intracranial aneurysm (*n* = 24)	CFU-EPC Migration to VEGF	Decreased proliferative and migratory capacity of EPC

Liu et al. [31]	Case-control TBI (*n* = 29)	CD34+ CD133+ in isolated PBMC	Decreased EPC in TBI, steady increase from day 5–7 with peak day 7

Liu et al. [32]	Case-control TBI (*n* = 84)	CD34+ CD133+ in isolated PBMC	(i) Decreased EPC 24–48 h after TBI, increase to day 7 (ii) Non-survivors lower EPC

Wei et al. [33]	Case-control ruptured cerebral aneurysm (*n* = 14)	CD34+ CD133+ Isolated PBMC	(i) Decreased number of EPC in patients (ii) Increase after coiling with a peak at day 14

Wei et al. [34]	Case-control cerebral aneurysm (*n* = 56, ruptured *n* = 35)	CD34+ CD133+ CD34+ KDR+ in isolated PBMC Migration to VEGF	(i) Both EPC phenotypes reduced in cerebral aneurysm (ii) Impaired migration and increased of EPC in cerebral aneurysm

EPC: endothelial progenitor cells; PBMC: peripheral blood mononuclear cells; VEGF: vascular endothelial growth factor; TBI: traumatic brain injury.

**Table 4:** Overview on endothelial microparticles in acute brain injury, SAH, and TBI.

Study group	Study population	Detection method	Phenotype EMP	Main findings
Lackner et al. [35]	Case-control spontaneous SAH (*n* = 20)	Flow cytometry plasma	CD105+/Annexin V+ or − CD62E+/Annexin V+ or − CD54+/Annexin V+ or − CD106+/Annexin V+ or −	(i) Increased number of all EMP phenotypes studied in SAH versus healthy (ii) In patients with Doppler detected cerebral vasospasm increased CD105+/Annexin V+ and CD62E+/Annexin V+ (iii) CD105+/Annexin V+ associated with cerebral infarction

Morel et al. [36]	Case-control TBI (*n* = 16)	Capture technique PFP and CSF	Annexin V+ CD31+	(i) Increased MP number in plasma and CSF at D0, decreased D3, D5, D10 (ii) High proportion of EMP

Sanborn et al. [37]	Case-control SAH (*n* = 22)	Flow cytometry Frozen plasma samples	CD146+/Annexin V+	(i) Elevated EMP after SAH, and remained high until D10 (ii) Negative correlation EMP and infarction at D14

CSF: cerebral spinal fluid; EMP: endothelial microparticles; MP: microparticle, PFP: Platelet free plasma; SAH: subarachnoid hemorrhage; TBI: traumatic brain injury.
